# Supplementary material for: Comparative multiomics analysis of cell physiological state after culture in a basket bioreactor
Source: Sci Rep. 2022 Nov 23;12:20161. doi: 10.1038/s41598-022-24687-4 (PMC9686226; doi:10.1038/s41598-022-24687-4)
Supplement: Supplementary file 1 — Supplementary Information 1. [file 41598_2022_24687_MOESM1_ESM.zip › raw data/Transcriptomic raw data/6.Enrichment/1.GO/TE1vsTC1/GSEA/TC1/LIPID_LOCALIZATION/LIPID_LOCALIZATION(GO_0010876).html]

Details for gene set LIPID\_LOCALIZATION(GO:0010876)[GSEA]


|  || Dataset | readcount.sample |
| Phenotype | sample.cls |
| Upregulated in class | TC1 |
| GeneSet | LIPID\_LOCALIZATION(GO:0010876) |
| Enrichment Score (ES) | -0.20711885 |
| Normalized Enrichment Score (NES) | -0.97485745 |
| Nominal p-value | 0.52134144 |
| FDR q-value | 0.85040194 |
| FWER p-Value | 1.0 |
Table: GSEA Results Summary

  

Fig 1: Enrichment plot: LIPID\_LOCALIZATION(GO:0010876)
  
Profile of the Running ES Score & Positions of GeneSet Members on the Rank Ordered List

  

| PROBE | DESCRIPTION   (from dataset) | GENE SYMBOL | GENE\_TITLE | RANK IN GENE LIST | RANK METRIC SCORE | RUNNING ES | CORE ENRICHMENT || 1 | 103229196 | LOC103229196 |  |  | 132 | Infinity | -0.0052 | Yes |
| 2 | 103226281 | PLEKHA8 |  |  | 2399 | 1.897 | 0.0348 | Yes |
| 3 | 103223245 | APOL1 |  |  | 2786 | 1.765 | 0.1488 | Yes |
| 4 | 103239093 | LOC103239093 |  |  | 10753 | 0.973 | -0.1348 | No |
| 5 | 103227754 | RFT1 |  |  | 11313 | 0.937 | -0.0900 | No |
| 6 | 103248547 | APOA1 |  |  | 11328 | 0.936 | -0.0210 | No |
| 7 | 103233919 | BSCL2 |  |  | 12187 | 0.882 | 0.0063 | No |
| 8 | 103228356 | LOC103228356 |  |  | 12423 | 0.868 | 0.0604 | No |
| 9 | 103223236 | APOL5 |  |  | 14049 | 0.766 | 0.0448 | No |
| 10 | 103245686 | FITM2 |  |  | 15089 | 0.702 | 0.0506 | No |
| 11 | 103236027 | LOC103236027 |  |  | 16236 | 0.625 | 0.0459 | No |
| 12 | 103225804 | LOC103225804 |  |  | 17148 | 0.562 | 0.0470 | No |
| 13 | 103234841 | APOE |  |  | 17432 | 0.539 | 0.0745 | No |
| 14 | 103218648 | APOLD1 |  |  | 17707 | 0.515 | 0.1005 | No |
| 15 | 103248542 | APOA5 |  |  | 19250 | 0.367 | 0.0589 | No |
| 16 | 103223235 | APOL6 |  |  | 19708 | 0.315 | 0.0619 | No |
| 17 | 103226860 | HILPDA |  |  | 19811 | 0.301 | 0.0798 | No |
| 18 | 103223244 | APOL2 |  |  | 20486 | 0.194 | 0.0641 | No |
| 19 | 103242201 | GLTPD2 |  |  | 20717 | 0.146 | 0.0646 | No |
| 20 | 103223625 | APOL4 |  |  | 20727 | 0.142 | 0.0748 | No |
| 21 | 119623720 | LOC119623720 |  |  | 21292 | 0.000 | 0.0496 | No |
Table: GSEA details
[plain text format]

  

Fig 2: LIPID\_LOCALIZATION(GO:0010876)
  
Blue-Pink O' Gram in the Space of the Analyzed GeneSet

  

Fig 3: LIPID\_LOCALIZATION(GO:0010876): Random ES distribution
  
Gene set null distribution of ES for
 **LIPID\_LOCALIZATION(GO:0010876)**
